# BBPpredict: A Web Service for Identifying Blood-Brain Barrier Penetrating Peptides

**DOI:** 10.3389/fgene.2022.845747

**Published:** 2022-05-17

**Authors:** Xue Chen, Qianyue Zhang, Bowen Li, Chunying Lu, Shanshan Yang, Jinjin Long, Bifang He, Heng Chen, Jian Huang

**Affiliations:** ^1^ Medical College, Guizhou University, Guiyang, China; ^2^ School of Life Science and Technology, University of Electronic Science and Technology of China, Chengdu, China

**Keywords:** blood-brain barrier, random forest (RF), nested cross-validation, computational method, blood-brain barrier penetrating peptides (BBPs)

## Abstract

Blood-brain barrier (BBB) is a major barrier to drug delivery into the brain in the treatment of central nervous system (CNS) diseases. Blood-brain barrier penetrating peptides (BBPs), a class of peptides that can cross BBB through various mechanisms without damaging BBB, are effective drug candidates for CNS diseases. However, identification of BBPs by experimental methods is time-consuming and laborious. To discover more BBPs as drugs for CNS disease, it is urgent to develop computational methods that can quickly and accurately identify BBPs and non-BBPs. In the present study, we created a training dataset that consists of 326 BBPs derived from previous databases and published manuscripts and 326 non-BBPs collected from UniProt, to construct a BBP predictor based on sequence information. We also constructed an independent testing dataset with 99 BBPs and 99 non-BBPs. Multiple machine learning methods were compared based on the training dataset via a nested cross-validation. The final BBP predictor was constructed based on the training dataset and the results showed that random forest (RF) method outperformed other classification algorithms on the training and independent testing dataset. Compared with previous BBP prediction tools, the RF-based predictor, named BBPpredict, performs considerably better than state-of-the-art BBP predictors. BBPpredict is expected to contribute to the discovery of novel BBPs, or at least can be a useful complement to the existing methods in this area. BBPpredict is freely available at http://i.uestc.edu.cn/BBPpredict/cgi-bin/BBPpredict.pl.

## 1 Introduction

Blood-brain barrier (BBB) highly protects the central nervous system (CNS) (Nance et al., 2022), preventing 98% of small molecules and 100% of large molecules from entering the brain (Sánchez-Navarro et al., 2017). It is the main obstacle for drug delivery into the brain (Banks, 2016). Therefore, exploring methods for drugs to penetrate BBB is a research hotpot in the development of drugs for CNS disorders (Terstappen et al., 2021).

Blood-brain barrier penetrating peptides (BBPs) can cross the BBB through various mechanisms without destroying the integrity of BBB (Van Dorpe et al., 2012; Oller-Salvia et al., 2016). It has been reported that partial BBPs can transfer drugs into the brain, which provides a new avenue for the development of drugs for CNS diseases (Zhou et al., 2021). Furthermore, because of their characteristics of easy synthesis, satisfactory effect, low toxicity and wide selectivity (Muttenthaler et al., 2021), BBPs show broad application prospects as carriers or therapeutic agents for CSN diseases treatment (Zhou et al., 2021). Nonaka et al. reported that IF7, an annexin A1-binding peptide, could overcome BBB and deliver chemotherapeutics to target brain tumors (Nonaka et al., 2020). Xie and coworkers demonstrated that d-peptide ligand of angiopep-2 modified nanoprobes could cross BBB and locate glioma sites (Xie et al., 2021). Lim and collaborators found that dNP2 peptide could penetrate BBB and deliver ctCTLA-4 protein to ameliorate autoimmune encephalomyelitis in mouse models (Lim et al., 2015). Kurzrock and Drappatz et al. showed that ANG1005 or GRN1005, a conjugate of angiopep-2 and paclitaxel, has reached clinical study for the treatment of glioma (Kurzrock et al., 2012; Drappatz et al., 2013).

There have been two BBP databases published to date, Brainpeps (Van Dorpe et al., 2012) and B3Pdb (Kumar et al., 2021b), since BBPs became candidates for developing peptide agents for managing CNS disorders. These studies are undoubtedly a strong boost to the development of medications for CNS diseases. However, the discovery of BBPs by wet-lab experiment is time-consuming and complex, and only hundreds of BBPs have been identified experimentally to date. Construction of computational methods for the identification of BBPs is very valuable for developing therapeutics for CSN diseases. Machine learning methods have been successfully applied to the classification of various peptides, such as cell-penetrating peptides (Wei et al., 2017a; Wei et al., 2017b; Kumar et al., 2018), antimicrobial peptides (Bhadra et al., 2018), anticancer peptides (Li and Wang, 2016). There are also two BBP predictors, BBPpred (Dai et al., 2021) and B3Pred (Kumar et al., 2021a), have published successively for identifying BBPs. BBPpred is based on logistic regression to identify BBPs, while B3Pred uses random forest (RF) to predict BBPs. Considering the low sample complexity of these two classifiers, the performance of computational models for identifying BBPs can be improved.

In this work, we collected more BBPs from existing databases (Van Dorpe et al., 2012; Kumar et al., 2021b) and published literatures to construct a new BBP predictor named BBPpredict, which is an online web service and freely available at http://i.uestc.edu.cn/BBPpredict/cgi-bin/BBPpredict.pl. By comparing the results of the nested five-fold cross-validation and independent testing dataset of various machine learning predictors, the RF-based model showed the best prediction performance. Thus, BBPpredict was implemented by using RF. We expect BBPpredict will help researchers find more novel BBPs.

## 2 Materials and Methods

### 2.1 Datasets

In this work, we selected experimentally validated BBPs as candidate positive samples that were collected from Brainpeps (Van Dorpe et al., 2012), B3Pdb(Kumar et al., 2021b), public datasets of BBPpred (Dai et al., 2021) and B3Pred (Kumar et al., 2021a), and other published literatures from PubMed with query “(((Brain [Title/Abstract]) OR (blood–brain barrier [Title/Abstract])) AND peptide [Title/Abstract]) AND (transport [Title/Abstract] OR transfer [Title/Abstract] OR permeation [Title/Abstract] OR permeability [Title/Abstract])”, covering the period 2011–2021. BBPs were then preprocessed as follows: 1) the repetitive sequences were eliminated; 2) peptide sequences with ambiguous residues (“X”, “B” and “Z”, etc.) were deleted (He et al., 2016). Finally, 425 BBPs were remained as positive samples. We also collected 1,304 non-BBPs that were obtained by the following three steps: 1) collect initial sequences from UniProt with the query “peptides length: [5 TO 50] NOT blood brain barrier NOT brain NOT brainpeps NOT b3pdb NOT permeation NOT permeability NOT venom NOT toxin NOT transmembrane NOT transport NOT transfer NOT membrane NOT neuro NOT hemolysis AND reviewed: yes” (Dai et al., 2021), 2) remove redundant sequences by using CD-HIT (sequence identity cut-off of 10%) (Dai et al., 2021), 3) exclude the peptide sequences with ambiguous residues (“X”, “B,” and “Z”, etc.).

### 2.2 Training and Independent Testing Datasets

To evaluate the performance of our predictor and existing predictors (BBPpred and B3Pred), 99 BBPs that collected through published literatures and 99 non-BBPs randomly selected from candidate negative samples construct an independent testing dataset that was completely independent of the training dataset of the three predictor models (BBPpred, B3Pred and our proposed BBPpredict) (Table 1). The remaining 326 BBPs were used as the positive training dataset. To balance the sample size for training, we randomly selected 326 non-BBPs as the negative training dataset (Table 1), whose length distribution is the same as the positive training dataset. All datasets are available for download from http://i.uestc.edu.cn/BBPpredict/download.html.

**TABLE 1:** List of training dataset and independent testing dataset.

Dataset	Number of BBPs	Number of Non-BBPs
Training dataset	326	326
Independent testing dataset	99	99

### 2.3 Feature Extraction

Feature extraction refers to the transformation of peptide sequences into fixed-length feature vectors, which is an indispensable step for the construction of predictors. In this study, we selected five feature encoding methods, including amino acid composition (AAC), dipeptide composition (DPC), composition of *k*-spaced amino acid group pairs (CKSAAGP, *k* = 3), pseudo-amino acid composition (PAAC) and grouped amino acid composition (GAAC) to extract the characteristics of peptide sequence. Here we set the length of a peptide to be *N*, and all feature extraction methods are based on 20 natural amino acids (i.e., “ACDEFGHIKLMNPQRSTVWY”). Feature extraction was implemented by an in-house script.

#### 2.3.1 Amino Acid Composition

AAC calculates the frequency of each amino acid in the peptide sequence (Bhasin and Raghava, 2004). It can be calculated as:

$$f\left(i\right)=\frac{N\left(i\right)}{N},i\in \left\{A,C,D,...Y\right\}$$ (1)

where *N(i)* is the number of the amino acid type *i*.

#### 2.3.2 Dipeptide Composition

DPC gives 400 descriptors (i.e.“ AA,AC,AD,…YY ”) (Saravanan and Gautham, 2015). It is defined as:

$$D\left(r,s\right)=\frac{N_{rs}}{N-1},r,s\in \left\{A,C,D,...Y\right\}$$ (2)

where *Nrs* is the number of the dipeptide consisting of amino acids *r* and *s* in the peptide sequence.

#### 2.3.3 Grouped Amino Acid Composition

For the GAAC encoding, 20 natural amino acids are firstly divided into five categories according to their physicochemical properties: amino acid groups g1 (GAVLMI), g2 (FYW), g3 (KRH), g4 (DE) and g5 (STCPNQ). Group g1 belongs to the aliphatic group, g2 aromatic group, g3 positive charge group, g4 negative charged group and g5 uncharged group, respectively. GAAC represents the frequency of each amino acid group (Lee et al., 2011) and can be described as:

$$\begin{array}{l} f\left(g\right)=\frac{N\left(g_{i}\right)}{N},i\in \left\{g1,g2,g3,g4,g5\right\}\\ N\left(g_{i}\right)=\sum N\left(i\right),i\in \left\{g1,g2,g3,g4,g5\right\} \end{array}$$ (3)

where $N\left(g_{i}\right)$ is the number of amino acids in group g, *N(i)* is the number of the amino acid type *i*.

#### 2.3.4 Composition of *K*-Spaced Amino Acid Group Pairs

CKSAAGP is based on CKSAAP (Chen et al., 2007a; Chen et al., 2007b, 2008; Chen et al., 2009) descriptor and GAAC descriptor, which calculates the frequency of *k*-spaced group pairs. And the detailed calculation of CKSAAGP can refer to (Chen et al., 2018). In this study, we set k as three by default. And when k = 0, CKSAAGP can be calculated as:

$$\left(\frac{N_{g1g1}}{N_{\mathrm{total}}},\frac{N_{g1g2}}{N_{\mathrm{total}}},\frac{N_{g1g3}}{N_{\mathrm{total}}},...\frac{N_{g1g5}}{N_{\mathrm{total}}}\right)25$$ (4)

Where $N_{total}$ describes *N-*1*,* $N_{gg}$ is the number of 0-spaced group pairs.

#### 2.3.5 Pseudo-Amino Acid Composition

PAAC describes the information of two residues order and properties in the peptide sequence. The computation of PAAC is available in (Chou, 2001; 2005).

After feature extraction, each peptide was encoded by a 550-dimensional feature vector, which was generated by concatenating five types of feature vector.

### 2.4 Feature Scoring and Selection

Generally, not all features make contribution to the model construction. Partial features make remarkable contributions, while some others make slight contributions (He et al., 2019). Therefore, feature selection is a very vital step for accomplishing a classifier model with promising classification performance (Zhao et al., 2016). In this study, F-score method was employed to estimate each feature’s contribution. The feature with a greater F-score implies its larger contribution for prediction model. We conducted the following procedures to select more informative features from the 550 features that were extracted from the training dataset. In the first stage, we evaluated the five-fold cross-validation performance of top 92, 184, 275, 367, 458, 550 features for various classification algorithms. In the five-fold cross-validation, the training dataset was equally divided into five subsets, among these five subsets, a subset was used as the testing-set and the other four subsets as the training-set. The division of top 92, 184, 275, 367, 458, 550 features based on the training-set was determined by making (count_max-count_min)/6 as the cut-off point of feature division, where “count_max” represents the maximum dimension of feature (550 features), and “count_min” is the minimum dimension of feature (1 feature). In the second stage, according to the five-fold cross-validation results of different classification algorithms, we obtained the number of features n with the highest accuracy. In the third stage, we selected top n features from the 550 features extracted from the training dataset and ranked by F-score in descending order to construct the final model.

### 2.5 Classification Model Construction

Eight traditional machine learning algorithms, including decision tree (DT), RF, k-nearest neighbors (KNN), adaptive boosting (AdaBoost), gentle adaptive boosting (GentleBoost), adaptive logistic regression (LogitBoost), linear support vector machine (linearSVM) and radial basis function (RBF) kernel SVM (rbfSVM) were used to build the predictive models based on the features selected by feature selection (see in Supplementary Table S3), respectively. LIBSVM 3.24 (http://www.csie.ntu.edu.tw/∼cjlin/libsvm/) was utilized to accomplish linearSVM and rbfSVM (Chang and Lin, 2011). DT, RF, KNN, AdaBoost, GentleBoost and LogitBoost are respectively implemented by MATLAB R2021a built-in functions fitcTree, TreeBagger, fitcknn and fitcEnmbles. To compare with deep learning method, a long-short term memory (LSTM) network that realized based on Keras 2.3.1 (tensorflow 2.1.0 as backend) package of python 3.6 was also utilized to construct the classification model (Hochreiter and Schmidhuber, 1997). The LSTM classification model consisted of one LSTM layer with eight hidden neurons. The non-linear activation function hyperbolic tangent (tanh) was applied to LSTM layer. It should be noted that for LSTM, the vectored sequence of peptide was utilized as classification features and no feature selection was applied. The pseudo code for final model construction can be found in the Supplementary Material.

### 2.6 Prediction Assessment

Five evaluation indexes, including accuracy (ACC), sensitivity (SN), specificity (SP), Matthews correlation coefficient (MCC) and the area under the receiver operating characteristic (ROC) curve (AUC), were utilized to quantify the performance of each predictive model. The first four indicators are calculated as follows:

$$SN=\frac{TP}{TP+FN}$$ (5)

$$SP=\frac{TN}{TN+FP}$$ (6)

$$ACC=\frac{TP+TN}{TP+FN+FP+TN}$$ (7)

$$MCC=\frac{TP\times TN-FP\times FN}{\sqrt{\left(TP+FP\right)\left(TP+FN\right)\left(TN+FP\right)\left(TN+FN\right)}}$$ (8)

where *TP* describes the number of genuine BBPs which are predicted as BBPs. *FN* represents the number of genuine BBPs that are identified as non-BBPs. Denote *TN* as the number of true non-BBPs classified as non-BBPs and *FP* the number of true non-BBPs identified as BBPs. *SN* and *SP* primarily assess the ability of a predictive model to identify positive and negative samples respectively, while *ACC* and *MCC* investigate the comprehensive capacity of a prediction model to classify both positive and negative samples (Wang et al., 2019). The AUC score is often utilized to judge the merits and demerits of classifiers. In this study, we selected the optimal predictive model according to the AUC value. The model construction and evaluation were performed at a computational server (Sugon I840-G20, Dawning Information Industry Co., LTD., Beijing, China).

### 2.7 Reproducible Analysis

Data analysis reproducibility plays a vital role for achieving an independent verification of the analysis results (Walzer and Vizcaíno, 2020). In this work, we constructed 100 testing datasets and corresponding training datasets to verify the robustness of the construction method of the BBP predictor. To avoid high similarity between the independent testing dataset and the testing dataset of the reproducible analysis, here each testing dataset consisted of 50 BBPs randomly selected from candidate positive samples (114 BBPs) that are independent of the training datasets of BBPpred and B3Pred and 50 non-BBPs with the same selection rules with BBPs. The model building process based on 100 reconstructed datasets for different classification algorithms (RF, rbfSVM, linearSVM, etc.) is consistent with the above method. The result of the reproducibility analysis can be found in the Supplementary Material.

## 3 Result

### 3.1 Overall Workflow

**FIGURE 1:**
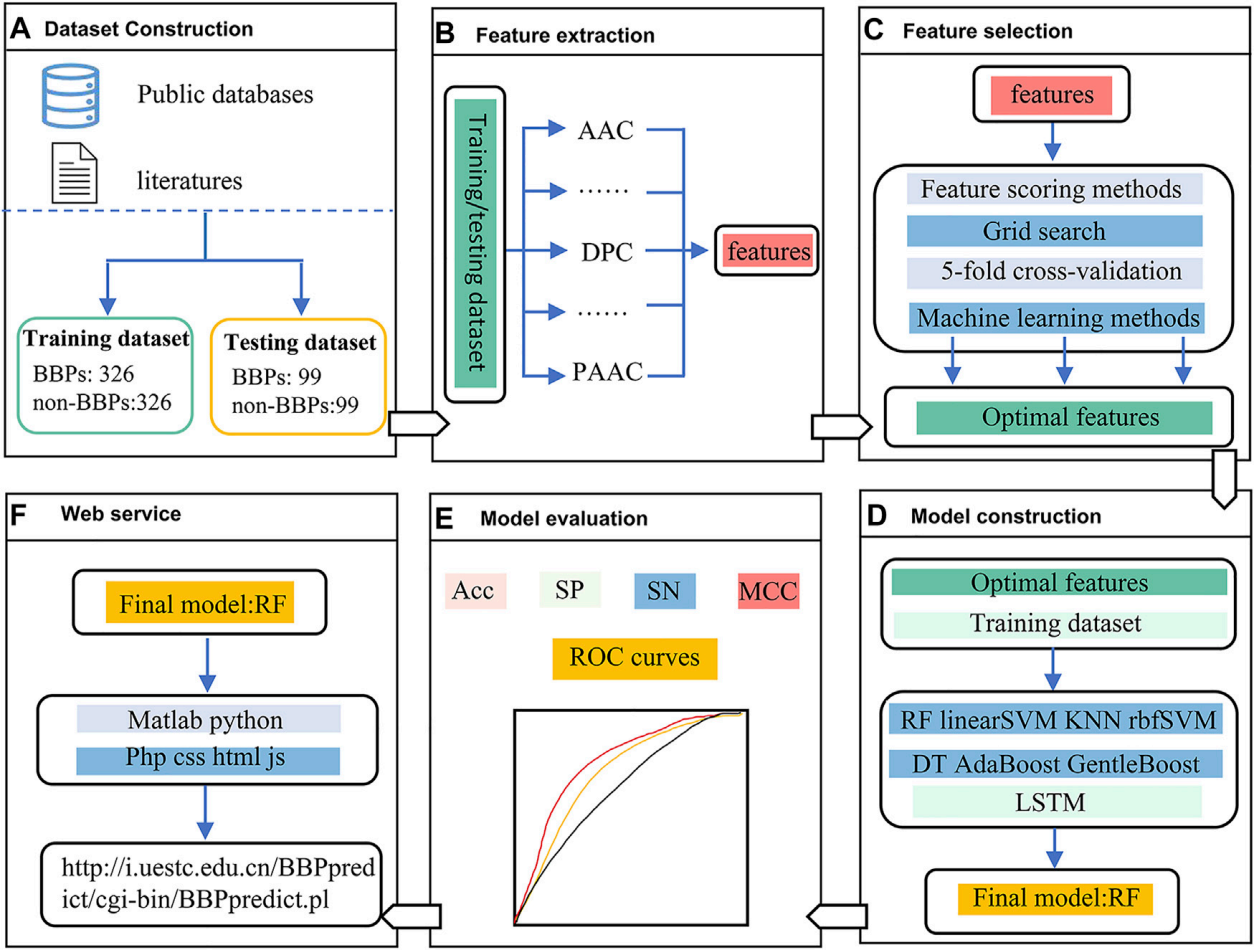
The framework of BBPpredict. **(A)**. Dataset Construction. **(B)**. Feature extraction. **(C)**. Feature selection. **(D)**. Model construction. **(E)**. Model evaluation. **(F)**. Web service.

The framework of this study is depicted in Figure 1. In the first stage, two benchmark datasets, including a training dataset and an independent testing dataset, were constructed. In the second stage, five feature extraction methods were utilized to encode each peptide sequence, and then a 550-dimensional feature vector was generated. In the third stage, feature scoring methods and grid search with five-fold cross-validation strategy was used for feature selection. In the fourth stage, multiple machine learning methods were employed to build different models. In the fifth stage, we evaluated the predictive performance of the nine models by using a nested five-fold cross-validation and an independent testing dataset, respectively. Finally, the RF model outperformed other models was selected as the final model, which was implemented into a web server.

### 3.2 Performance of Nine Classifiers in Nested Five-Fold Cross-Validation

**FIGURE 2:**
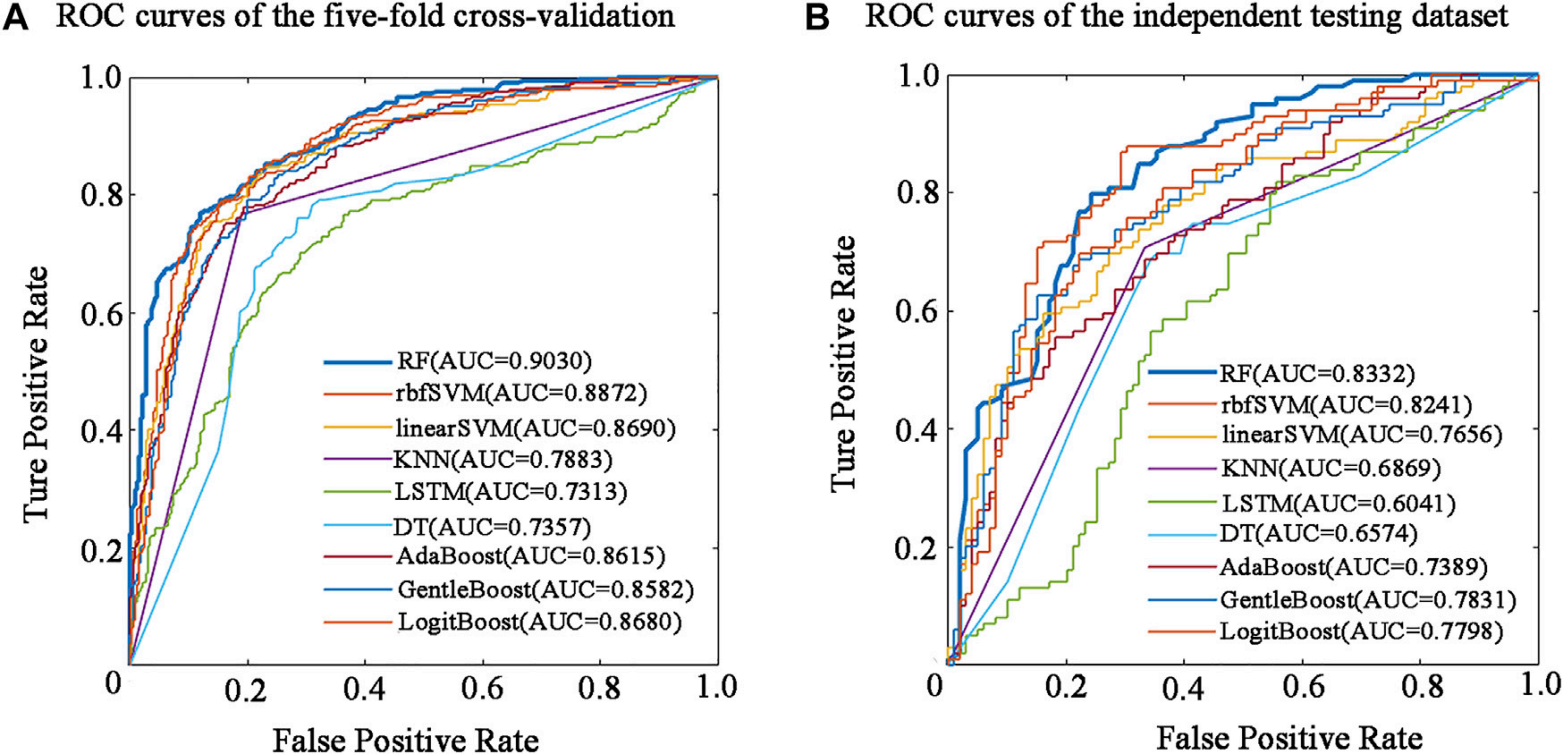
Performance evaluation of different predictors in five-fold cross-validation and independent testing dataset. **(A)** ROC curves of the five-fold cross-validation. **(B)** ROC curves of the independent testing dataset.

The performance of the nine predictive models in the nested five-fold cross-validation is shown in Table 2, and the ROC curves are illustrated in Figure 2A. For a detailed description of nested five-validation cross-validation, please refer to the Supplementary Material. In Table 2, RF model outperformed the other eight machine learning models. All five evaluation metrics reached the highest level. It has an AUC score of 0.9030, ACC value of 81.90%, MCC value of 0.6390, SN value of 79.14% and SP value of 84.66% (see Table 2). Moreover, compared with the eight conventional machine learning classifiers, the performance of LSTM is not satisfactory. Except for SP, the values of the other four evaluation metrics of LSTM model were the lowest. The overall performance of traditional machine learning algorithms is generally better than LSTM.

**TABLE 2:** The prediction performances of different classifiers in nested five-fold cross-validation.

Scoring Method	Classifier	SN(%)	SP(%)	ACC(%)	MCC	AUC
F-score	**RF**	**79.14**	**84.66**	**81.90**	**0.6390**	**0.9030**
	KNN	76.69	80.98	78.83	0.5772	0.7883
	rbfSVM	78.83	83.13	80.98	0.6202	0.8872
	linearSVM	75.77	83.13	79.45	0.5906	0.8690
	DT	71.78	74.54	73.16	0.4634	0.7357
	LSTM	65.23	75.38	70.31	0.4083	0.7313
	AdaBoost	77.91	80.67	79.29	0.5861	0.8615
	GentleBoost	77.30	80.06	78.68	0.5738	0.8582
	LogitBoost	79.14	82.21	80.67	0.6138	0.8680

### 3.3 Performance of Nine Classifiers on the Independent Testing Dataset

To determine the final model for constructing BBPpredict, performance evaluation on the independent testing dataset is much more convincing than five-fold cross-validation. According to the steps in the method section, nine classification models are established by using the training dataset. The independent testing dataset was then utilized to test the performance of these models. As depicted in Table 3 and Figure 2B, in term of AUC score, the RF model also performed best, with a score of 0.8332, higher than rbfSVM, linearSVM, KNN, DT, GentleBoost, AdaBoost, LogitBoost and LSTM classifiers by 0.0091, 0.0676, 0.1463, 0.1758, 0.0501, 0.0943, 0.0534 and 0.2291 respectively. In terms of accuracy and MCC, the RF classifier also achieved impressive values, with scores of 77.27% and 0.5455, which are better than other eight classifier algorithm predictors. Furthermore, the LSTM classifier had the weakest generalization ability. In addition, results of the reproducibility analysis for nine classifiers are highly consistent with the above results (see Supplementary Table S9).

**TABLE 3:** The prediction performances of different classifiers in the independent testing dataset.

Scoring Method	Classifier	SN(%)	SP(%)	ACC(%)	MCC	AUC
F-score	**RF**	**76.77**	**77.78**	**77.27**	**0.5455**	**0.8332**
	rbfSVM	78.79	73.74	76.26	0.5259	0.8241
	KNN	70.71	66.67	68.69	0.3740	0.6869
	DT	69.70	61.62	65.66	0.3142	0.6574
	linearSVM	64.65	74.75	69.70	0.3960	0.7656
	LSTM	58.59	63.64	61.11	0.2225	0.6041
	AdaBoost	64.65	68.69	66.67	0.3336	0.7389
	GentleBoost	74.75	66.67	70.71	0.4155	0.7831
	LogitBoost	67.68	77.78	72.73	0.4569	0.7798

### 3.4 Performance of the Predictions Under the Combinations of RF With Three Feature Scoring Methods

We also used the RF algorithm with optimal features selected by Pearson and Lasso feature scoring methods to construct prediction model. As shown in Supplementary Tables S4,5, the model under the combination of RF and F-score achieved the second highest AUC value in the nested five-fold cross-validation and the highest AUC value in the independent testing dataset. Therefore, we finally chose the combination of RF and F-score to build the final model based on 184 features and tree depth of 63.

### 3.5 Prediction Performance of Existing Predictors

There are two published predictors for identifying BBPs, B3Pred and BBPpred. These predictors and our predictor are based on peptide sequence information. The comparison of datasets of existing predictors and our proposed predictor can be seen in Table 4 (Detailed comparison can be found in Supplementary Table S8). To be fair, an independent testing dataset, which is completely independent of three predictors’ training datasets, was used to compare their performance. As shown in Table 5, compared with the existing BBPs predictors, our predictor achieved a promising performance (ACC = 77.27%, SN = 76.77%, SP = 77.78% and MCC = 0.5455), it outperformed BBPpred and B3Pred, higher than them by 10.6% and 9.59% in accuracy, severally, with MCC increasing 0.2121 and 0.1913, respectively. There were remarkable improvements in sensitivity and specificity (see Table 5). The above results demonstrate that BBPpredict is more capable of distinguishing between BBPs and non-BBPs than BBPpred and B3Pred.

**TABLE 4:** Comparison of datasets for three predictors.

	BBPpred	B3Pred	BBPpredict
Data source	Positive: Brainpeps, PepBank, articles, SATPdb	Positive: B3Pdb	Positive: Brainpeps, B3Pdb, BBPpred, B3Pred, articles
	Negative: UniProt	Negative: UniProt	Negative: UniProt
Article search deadline		22 July 2020	Nov. 2021
Article number	7	271	300
Positive sample number	119 (training:100, testing: 19)	269 (training:215, testing: 54)	425 (training:326, testing: 99)
Negative sample number	119 (training:100, testing: 19)	2,690 (training: 2,152, testing:538)	425 (training:326, testing: 99)
Peptide length	5–50	6–30	5–50

**TABLE 5:** The prediction performances of different predictors.

Predictor	SN(%)	SP(%)	ACC(%)	MCC
**BBPpredict**	**76.77**	**77.78**	**77.27**	**0.5455**
BBPpred	67.68	65.66	66.67	0.3334
B3Pred	70.71	64.65	67.68	0.3542

### 3.6 Web Server Implementation

**FIGURE 3:**
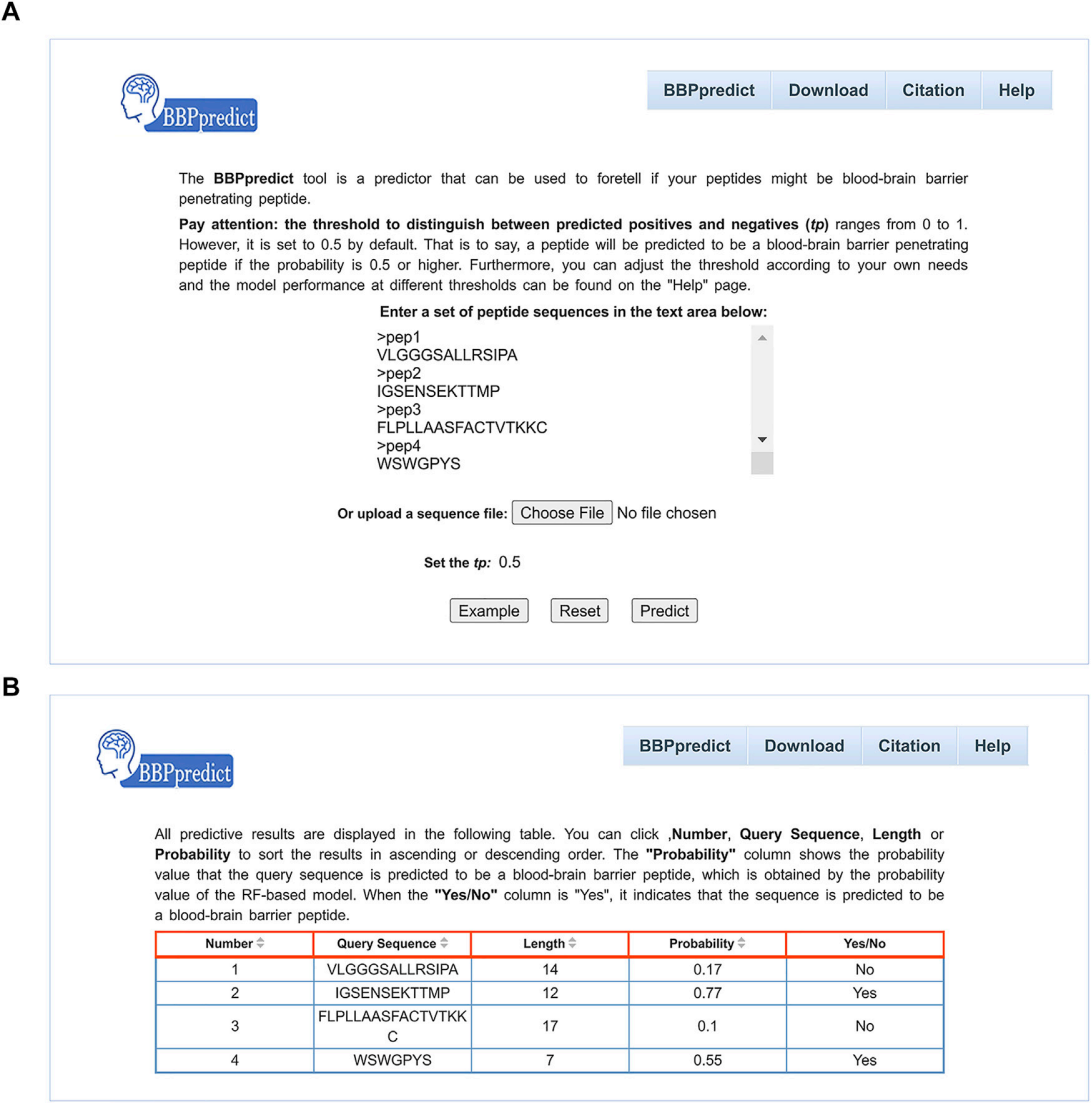
Web interface of BBPpredict. **(A)** The query sequences and threshold of the probability value (tp) are required to be submitted in the input interface. **(B)** The result page returned from BBPpredict.

To facilitate users to identify BBPs, we established an online web service named BBPpredict that was implemented based on optimized features and the RF model. BBPpredict can be accessed at http://i.uestc.edu.cn/BBPpredict/cgi-bin/BBPpredict.pl, conveniently. The web service of BBPpredict was developed by using Perl and Html, *Python* and Matlab. Users can paste peptide sequences or upload a sequence file to predict BBPs, as illustrated in Figure 3A. Then click the “Predict” button to make predictions, and the predictive results are depicted in Figure 3B.

BBPpredict allows users to adjust the threshold of the probability value (tp) to distinguish between predicted positives and negatives, which can range from 0 to 1. As shown in Table 6, with the increase of tp, the value of SN decreases, and the SP increases. When tp is 0.5, ACC achieves the highest score of 77.27%, MCC reaches the highest value of 0.5455.

**TABLE 6:** Performance of BBPpredict in the independent testing dataset when tp changes.

tp	SN (%)	SP (%)	ACC (%)	MCC
0.1	100	11.11	55.56	0.2425
0.2	98.99	29.29	64.14	0.3944
0.3	94.95	44.44	69.70	0.4564
0.4	86.87	64.65	75.76	0.5284
0.5	76.77	77.78	77.27	0.5455
0.6	58.59	82.83	70.71	0.4269
0.7	45.45	90.91	68.18	0.4082
0.8	36.36	96.97	66.67	0.4191
0.9	13.13	97.98	55.56	0.2100
0.95	5.05	97.98	51.51	0.0820

## 4 Discussion

In the past 30 years, many studies have demonstrated that BBPs are promising for the treatment of CNS diseases. BBPs can pass through the BBB and enter brain parenchyma without destroying BBB. Them can be used as transport carriers of DNA, RNA and protein as well as drug-assisted treatment and diagnosis of CNS diseases. However, the discovery of BBPs is still a thorny problem. Only a few hundreds of peptides have been experimentally confirmed as BBPs so far, since BBPs were discovered in 1996 (Banks and Kastin, 1996). Therefore, to facilitate the treatment of CNS diseases, it is necessary to employ computational methods to rapidly discover and identify more novel BBPs.

At present, two BBPs predictors, BBPpred (Dai et al., 2021) and B3Pred (Kumar et al., 2021a), have been proposed. Compared with these two predictors, our developed BBPpredict tool was based on a larger training dataset (as shown in Table 4). Besides the difference of the training dataset, a nested cross-validation strategy was utilized in the construction of BBPpredict. For common cross-validation, the model parameters were determined manually, and the accuracy based on the cross-validation would be affected by the artificial selection of model parameters, which usually overestimate the accuracy based on the cross-validation. For nested cross-validation, the model parameters were determined automatically. We speculated that this might be a reason why the previous two predictors had better performance in the cross-validation but had poor performance in our independent testing dataset. BBPpredict showed a large improvement in performance with nearly 6% sensitivity, 12% specificity, 10% accuracy and 0.20 MCC increase, compared with BBPpred and B3Pred. The elevated performance can save cost for researchers to identify BBPs and speed up the discovery of BBPs.

The BBPpredict website allows users to set the tp value. We tested the performance of BBPpredict in the independent testing dataset and provided sensitivity and specificity values under different tp values, which can serve as reference for users and increases the confidence they can have about the positive predictions.

We also reconstructed the BBPs/non-BBPs classification models with different machine learning methods using the new feature vectors that were generated from 16 feature extraction methods, including AAC, DPC, CKSAAGP, PAAC, GAAC, Grouped Di-Peptide Composition (GDPC) (Chen et al., 2018; Chen et al., 2020), Dipeptide Deviation from Expected Mean (DDE) (Chen et al., 2020), Composition (CTDC) (Dubchak et al., 1995; Dubchak et al., 1999; Chen et al., 2020), Transition (CTDT) (Dubchak et al., 1995; Dubchak et al., 1999; Chen et al., 2020), Distribution (CTDD) (Chen et al., 2020), Amphiphilic Pseudo-Amino Acid Composition (APAAC) (Chou, 2005; Jiao and Du, 2016), Quasi-sequence-order (QSOrder) (Chen et al., 2020), Normalized Moreau-Broto Autocorrelation (NMBroto) (Chen et al., 2018), Geary correlation (Geary) (Chen et al., 2020), Moran correlation (Moran) (Feng and Zhang, 2000; Chen et al., 2020) and Sequence-Order-Coupling Number (SOCNumber) (Lim et al., 2015). The detailed description of the last 11 feature encoding approaches can be found in the Supplementary Materials. F-score was used for feature sorting, grid search with five-fold cross-validation was utilized to select the best feature parameters and the best classifier parameters for different classifiers. Supplementary Tables S6,7 illustrated the detailed results of five-fold cross-validation and independent testing dataset of reconstructed classification models, respectively. However, the addition of feature encoding methods did not improve the classification performance of the model. We speculate that it is caused by the high correlation between the extracted features based on different feature extracting methods, which might induce highly correlated features in the final feature subset. As the feature number is limited, the highly correlated features might reduce useful information for model construction. Another possible reason might be the limited sample size, which might cause high false positive rate during the process of feature selection. The increase of feature size would lead to the increase of false positive features, which would affect the robustness of the predictive model.

BBPs pass through BBB via six penetration mechanisms, including diffusion transport, carrier-mediated transcytosis, efflux transporter, receptor-mediated transcytosis, adsorptive-mediated transcytosis and cell-mediated transcytosis (Zhou et al., 2021). The abilities of BBPs to penetrate BBB vary depending on their penetration mechanisms (Sánchez-Navarro et al., 2017). Therefore, we speculate the differences in their penetration mechanisms may affect the reliability of screening in the procession of model construction. However, BBPs of distinct penetration mechanisms were not further divided when constructing the positive sample of BBPpred, B3Pred and BBPpredict, because the number of BBPs for a specific transport mechanism is insufficient to construct a BBP predictor.

In the present work, we utilized RF algorithm to construct BBP predictor. The RF is an ensemble algorithm which is composed of several weak classifiers (decision trees). Our constructed model contains 63 decision trees. We speculate that these different decision trees might cover different penetration mechanisms and it might be the reason why the RF algorithm is superior to other machine learning algorithms. In the future, if the number of BBPs with a certain transport mechanism increase, it is possible and preferable to construct new BBP predictors using BBPs with the same penetrating mechanism.

## 5 Conclusion

In this study, we proposed an RF-based predictor for identifying BBPs, called BBPpredict, which is available for free at http://i.uestc.edu.cn/BBPpredict/cgi-bin/BBPpredict.pl. To find the optimal classifier, eight traditional machine learning algorithms and one deep learning algorithm were used for developing models. The RF algorithm was selected to construct BBPpredict after comparing the results of nine classifiers in the five-fold cross-validation and independent test. The RF-based model reached an AUC of 0.9030 with an accuracy of 81.90% and an AUC of 0.8332 with an accuracy of 77.27% in the nested five-fold cross-validation and independent testing dataset, respectively. We also compared BBPpredict with two existing BBPs predictors, BBPpred and B3Pred. The results showed that BBPpredict was remarkably higher in accuracy, MCC, sensitivity and specificity than these two predictors. BBPpredict is a promising classification model, and we expect it to play a positive role in the discovery of BBPs to facilitate the development of drugs for CNS diseases.

## Data Availability

The original contributions presented in the study are included in the article/Supplementary Material, further inquiries can be directed to the corresponding authors.
