# Supplementary material for: BBPpredict: A Web Service for Identifying Blood-Brain Barrier Penetrating Peptides
Source: Front Genet. 2022 May 17;13:845747. doi: 10.3389/fgene.2022.845747 (PMC9152268; doi:10.3389/fgene.2022.845747)
Supplement: Supplementary file 1 [file DataSheet1.pdf]

# *Supplementary Material*

## 1. Descriptions of feature descriptors for peptide sequences

Here we set the length of a peptide to be *N*, and all feature extraction methods are based on 20 natural amino acids (i.e., "ACDEFGHIKLMNPQRSTVWY"). Feature extraction was implemented by an in-house script.

### 1.1 Composition/Transition/Distribution (CTD)

Feature descriptors of CTD represent the amino acid distribution models of particular physicochemical property or structural in a peptide or protein sequence (Dubchak et al., 1995; Dubchak et al., 1999; Cai et al., 2003; Cai et al., 2004). 13 kinds of physicochemical properties have been used to calculate these features, including hydrophobicity, solvent accessibility, charge, secondary structures, polarity, and normalized Van der Waals Volume. For the detailed process of feature extractions, please refer to (Chen et al., 2018).

#### 1.1.1 CTDC

CTDC describes the composition of each amino acid, which consists of three values: the percentage of hydrophobic, polar and neutral residues of the protein and can be defined as follows:

$$C(r) = \frac{N(r)}{N}, r \in \{polar, neutral, hydrophobic\} \quad (1)$$

where $N(r)$ describes the number of amino acid type r in the sequence.

#### 1.1.2 CTDT

CTDT describes the frequency of amino acid combined with another amino acids residues, which also consists of three values. It is given as

$$T(r,s) = \frac{N(r,s)+N(s,r)}{N-1}, r,s \in \{(polar, neutral), (neutral, hydrophobic), (hydrophobic, polar)\} \quad (2)$$

where $N(r,s)$ and $N(s,r)$ are numbers of dipeptides as 'rs' and 'sr' in the sequence, respectively.

#### 1.1.3 CTDD

CTDD consists of five values for each of the three groups (polar, neutral and hydrophobic). The details of CTDD features can be available in (Dubchak et al., 1995; Dubchak et al., 1999; Chen et al., 2018).

### 1.2 Dipeptide Deviation from Expected Mean (DDE)

DDE feature vector is constructed by the following three parameters(Saravanan and Gautham, 2015).

$D_c(r,s)$, the frequency of dipeptide 'rs' in sequence, is given as

$$D_c(r,s) = \frac{N_{rs}}{N-1}, r,s \in \{A,C,D,...Y\} \quad (3)$$

where $N_{rs}$ is the number of the dipeptide consisting of amino acids $r$ and $s$ in the peptide sequence.

$T_m(r,s)$, the theoretical mean, is given by:

$$T_m(r,s) = \frac{C_r}{C_N} \times \frac{C_S}{C_N} \quad (4)$$

where $C_r$ represents the number of codons that code for amino acid $r$ in dipeptide '$rs$' and $C_S$ represents the number of codons which code for amino acid $s$ in dipeptide '$rs$'. $C_N$ is the number of all possible codons excluding the three stop codons.

$T_v$ ($r,s$), the theoretical variance of the dipeptide '$rs$', is defined as:

$$T_V(r,s) = \frac{T_m(r,s)(1-T_m(r,s))}{N-1} \quad (5)$$

Finally, $DDE(r,s)$ is given by:

$$DDE(r,s) = \frac{D_c(r,s) - T_m(r,s)}{\sqrt{T_V(r,s)}} \quad (6)$$

**1.3 Grouped Di-Peptide Composition (GDPC)**

The GDPC encoding is similar to DPC descriptor. It is composed of a total of 25 descriptors, which can be calculated as:

$$f(r,s) = \frac{N_{rs}}{N-1}, r,s \in \{g1, g2, g3, g4, g5\} \quad (7)$$

where $N_{rs}$ is the number of amino acid type groups $r$ accompanied by and type groups $s$. g1, g2, g3, g4 and g5 represent amino acid groups (GAVLMI), (FYW), (KRH), (DE) and (STCPNQ), respectively.

**1.4 Moran correlation (Moran)**

The Moran feature is described according to the distribution of amino acid properties in peptides or protein sequence(Horne, 1988; Feng and Zhang, 2000; Sokal and Thomson, 2006; Xiao et al., 2015). The amino acid properties are descripted based on different types of amino acids index that can be accessed at http://www.genome.jp/dbget/aaindex.html/.The computation of Moran is available in (Chen et al., 2018).

**Geary correlation (Geary)**

Geary is also a features descriptor that describes the properties of amino acids for a protein or peptide sequence (Sokal and Thomson, 2006; Chen et al., 2018). It can be calculated as:

$$C(d)=\frac{\frac{1}{2(N-d)}\sum_{i=1}^{N-d}(P_i-P_{i+d})^2}{\frac{1}{N-1}\sum_{i=1}^{N}(P_i-\overline{P'})^2},d=1,2,...,nlag \quad (8)$$

Where d represents the lag of the autocorrelation, *nlag* is the maximum value of the lag (default value:30), $P_i$ is the properties of the amino acids at positions *i*, $P_{i+d}$ is the properties of the amino acids at positions *i+d*. $\overline{P'}$ is average of the considered property P over the entire sequence, it can be calculated as:

$$\overline{P'}=\frac{\sum_{i=1}^{N}P_i}{N} \quad (9)$$

**1.5 Normalized Moreau-Broto Autocorrelation (NMBroto)**

The MBroto descriptors (Horne, 1988) are defined as follows:

$$AC(d)=\sum_{i=1}^{N-d}P_i\times P_{i+d},d=1,2,...,nlag \quad (10)$$

The normalized descriptors are thus calculated as:

$$ATS(d)=\frac{AC(d)}{N-d},d=1,2,...,nlag \quad (11)$$

where definitions of *d*, $P_i$ and $P_{i+d}$ are consistent with the description above.

**1.6 SOCNumber (Sequence-Order-Coupling Number)**

The *d*-th rank sequence-order-coupling number is calculated as:

$$\tau_d=\sum_{i=1}^{N-d}(d_{i,i+d})^2,d=1,2,3,...nlag \quad (12)$$

where $d_{i,i+d}$ is the entry in a given distance matrix describing a distance between the amino acids at position *i* and the amino acids at position *i + d, nlag* has the same definitions with the description above.

**1.7 QSOrder (Quasi-sequence-order)**

A quasi-sequence-order descriptor can calculate for each amino acid type, it defined as:

$$X_r=\frac{f_r}{\sum_{r=1}^{20}f_r+\sum_{d=1}^{nlag}\tau_d},r=1,2,....,20 \quad (13)$$

where $f_r$ represents the normalized occurrence of amino acid type r, there are same definitions as described above of nlag and $\tau_d$ . $X_r$ represents the first 20 quasi-sequence-order descriptors. The other 30 quasi-sequence-order descriptors are calculated as:

$$X_d = \frac{w\tau_d - 20}{\sum_{r=1}^{20} f_r + w\sum_{d=1}^{nlag} \tau_d}, d = 21, 22, ...20 + nlag \tag{14}$$

where $w$ is a weighting factor ($w$ = 0.1).

### 1.8 APAAC (Amphiphilic Pseudo-Amino Acid Composition)

APAAC was proposed in (Chou, 2005; Jiao and Du, 2016), which is like the PAAC descriptors. The details of APAAC features can be found in (Chou, 2001; Chen et al., 2018).

In this study, 1428 features can be obtained from the BBP/non-BBP sequence finally.

## 2. Nested cross validation

A nested five-fold cross-validation was applied on the training dataset (326 BBPs and 326 non-BBPs) to evaluate the prediction performance. Nested cross-validation has an inner and outer loop. The inner loop serves for model/parameter selection, while the outer loop is responsible for estimating the quality of the models trained in the inner layer. In this work, the training dataset (326 BBPs and 326 non-BBPs) was equally divided into five subsets in the outer layer. Among these five subsets, a subset was used as the testing-set and the other four subsets as the training-set. In the inner loop, the data of the training-set constructed in the outer layer were regrouped into five subsets of the same size, where four subsets were employed for tuning parameters (feature number and classifier parameters, details could be found in Tables S1 and S2), and one for evaluating models. It should be noted that the F-scores were calculated based on the training-set of the inner loop.

## 3. Pseudo code of nested cross validation and final model construction

### 3.1 Pseudo code of the nested cross validation

```
parameter_combinations = grid_search(feautre_selection_parameters, classifier_parameters)
for i = 1:5
    data_test_cv_outer = data_whole{i}
    data_train_cv_outer = data_whole-data_test_cv_outer
    for k = 1:number_of_parameter_combinations
        for j = 1:5
            data_test_cv_inner = data_train_cv_outer{j}
            data_train_cv_inner = data_train_cv_outer-data_test_cv_inner
            feature_selected_inner =
```

```
Fscore(data_train_cv_inner,feature_selection_parameters{k})
                classifer_inner = classifier_construct(feature_selected_inner, classifier_parameters{k})
                label_predict_cv{j} = predict(classifer_inner,data_test_cv_inner)
            end
            acc_cv(k) = acc_calculate(label_actual_cv,label_predict_cv)
    end
    index_max_acc_cv = max_index(acc_cv)
    best_parameter = parameter_combinations{index_max_acc_cv)
    feature_selected_outer = Fscore(data_train_cv_outer,best_feature_selection_parameter)
    classifier_outer = classifier_construct(feature_selected_outer,best_classifier_parameter)
    label_predict{i} = predict(classifier_outer,data_test_cv_outer)
end
acc_final = acc_calculate(label_actual,label_predict)
```

**3.2 Pseudo code of the final model construction**

```
parameter_combinations = grid_search(feautre_selection_parameters, classifier_parameters)
for i = 1:5
    data_test_cv_outer = data_whole{i}
    data_train_cv_outer = data_whole-data_test_cv_outer
    for k = 1:number_of_parameter_combinations
        for j = 1:5
            data_test_cv_inner = data_train_cv_outer{j}
            data_train_cv_inner = data_train_cv_outer-data_test_cv_inner
            feature_selected_inner = Fscore(data_train_cv_inner,feature_selection_parameters{k})
            classifer_inner = classifier_construct(feature_selected_inner, classifier_parameters{k})
            label_predict_cv{j} = predict(classifer_inner,data_test_cv_inner)
        end
        acc_cv(k) = acc_calculate(label_actual_cv,label_predict_cv)
    end
    index_max_acc_cv = max_index(acc_cv)
    best_parameter = parameter_combinations{index_max_acc_cv)
    feature_selected_outer = Fscore(data_train_cv_outer,best_feature_selection_parameter)
    classifier_outer = classifier_construct(feature_selected_outer,best_classifier_parameter)
    label_predict{i} = predict(classifier_outer,data_test_cv_outer)
end
acc_final = acc_calculate(label_actual,label_predict)
```

4. **Result of the reproducibility analysis**

The results of the reproducible analysis are listed in Table S9. In Table S9, the accuracy, MCC, AUC, sensitivity and specificity of 100 data-sets based on RF algorithm are 76.25%±3.56%, 0.5264±0.0710, 0.8563±0.0309, 75.36%±5.54% and 77.14%±4.62%, respectively. These results are highly consistent with the results in Table 3.

5. **Supplementary Tables**

Table S1. Parameters in the feature selection

| | | | | | | |
|---|---|---|---|---|---|---|
| Feature number | 92 | 184 | 275 | 367 | 458 | 550 |

Table S2. Model parameters of different classifiers

| Classifier | Model parameter | | | | | | |
|---|---|---|---|---|---|---|---|
| RF | Tree depth | 1 | 3 | 15 | 63 | 251 | 1000 |
| KNN | k-value | 1 | 2 | 3 | 4 | 5 | 6 |
| linearSVM | g | 1.0000e-04 | 0.0025 | 0.0631 | 1.5849 | 39.8170 | 1000 |
| rbfSVM | c | 1.0000e-05 | 3.9811e-04 | 0.0158 | 0.6310 | 25.1189 | 1000 |
| | g | 1.0000e-05 | 3.9811e-04 | 0.0158 | 0.6310 | 25.1189 | 1000 |
| DT | / | / | / | / | / | / | / |
| AdaBoost | / | / | / | / | / | / | / |
| GentleBoost | / | / | / | / | / | / | / |
| LogitBoost | / | / | / | / | / | / | / |

Table S3. Parameters for final model construction

| Classifier | Feature number | Model parameter |
|---|---|---|
| RF | 184 | Tree depth = 63 |
| KNN | 275 | k-value = 1 |
| linearSVM | 550 | g=1.5849 |
| rbfSVM | 367 | c=25.119, g=0.6310 |
| DT | 275 | / |
| AdaBoost | 550 | / |
| GentleBoost | 184 | / |
| LogitBoost | 275 | / |

Table S4. Performance of the predictions under the combinations of RF with three feature scoring methods based on five-fold cross-validation

| Machine learning method | Feature scoring method | SN(%) | SP(%) | ACC(%) | MCC | AUC |
|---|---|---|---|---|---|---|
| RandomForest | Fscore | 79.14 | 84.66 | 81.90 | 0.6390 | 0.9030 |
| | Pearson | 79.14 | 83.13 | 81.13 | 0.6232 | 0.9046 |
| | Lasso | 78.83 | 83.74 | 81.29 | 0.6265 | 0.8978 |

Table S5. Performance of the predictions under the combinations of RF with three feature scoring methods based on independent testing dataset

| Machine learning method | Feature scoring method | SN(%) | SP(%) | ACC(%) | MCC | AUC |
|---|---|---|---|---|---|---|
| RandomForest | Fscore | 76.77 | 77.78 | 77.27 | 0.5455 | 0.8332 |
| | Pearson | 72.73 | 73.74 | 73.23 | 0.4647 | 0.8276 |
| | Lasso | 67.68 | 79.80 | 73.74 | 0.4783 | 0.8276 |

Table S6. The prediction performances of different classifiers based on five-fold cross-validation

| Number of feature descriptors | Classifier | SN(%) | SP(%) | ACC(%) | MCC | AUC |
|---|---|---|---|---|---|---|
| sixteen | RF | 80.06 | 82.52 | 81.29 | 0.6260 | 0.9019 |
| | KNN | 68.71 | 78.83 | 73.77 | 0.4779 | 0.8067 |
| | rbfSVM | 67.79 | 74.23 | 71.01 | 0.4211 | 0.7898 |
| | linearSVM | 78.22 | 79.14 | 78.68 | 0.5736 | 0.8496 |
| | DT | 69.95 | 72.09 | 71.01 | 0.4203 | 0.7085 |
| | LSTM | 65.23 | 75.38 | 70.31 | 0.4083 | 0.7313 |
| | AdaBoost | 78.53 | 79.75 | 79.14 | 0.5829 | 0.8742 |
| | GentleBoost | 78.53 | 80.06 | 79.29 | 0.5860 | 0.8687 |
| | LogitBoost | 78.53 | 80.98 | 79.75 | 0.5953 | 0.8744 |

Table S7. The prediction performances of different classifiers based on independent testing dataset

| Number of feature descriptors | Classifier | SN(%) | SP(%) | ACC(%) | MCC | AUC |
|---|---|---|---|---|---|---|
| sixteen | RF | 74.45 | 76.77 | 75.76 | 0.5153 | 0.8414 |
| | rbfSVM | 69.70 | 77.78 | 73.74 | 0.4763 | 0.7783 |
| | KNN | 58.59 | 75.76 | 67.17 | 0.3486 | 0.6717 |
| | DT | 73.74 | 60.61 | 67.17 | 0.3464 | 0.6797 |
| | linearSVM | 64.65 | 71.72 | 68.18 | 0.3645 | 0.7306 |

| | | | | | |
|---|---|---|---|---|---|
| | LSTM | 58.59 | 63.64 | 61.11 | 0.2225 | 0.6041 |
| | AdaBoost | 71.72 | 69.70 | 70.71 | 0.4142 | 0.7810 |
| | GentleBoost | 73.74 | 68.69 | 71.21 | 0.4248 | 0.7792 |
| | LogitBoost | 75.76 | 74.75 | 75.25 | 0.5051 | 0.8008 |

Table S8. The data sources of three predictors

| | BBPpred | B3Pred | BBPpredict |
|---|---|---|---|
| Data source | Positive: Brainpeps, Pepbank, articles, STAPdb | Positive: B3Pdb | Positive: Brainpeps, B3Pdb, BBPpred, B3Pred, articles |
| | Negative: UniPort | Negative: UniPort | Negative: UniPort |
| Article search rules | none | PubMed: 'blood–brain barrier' or 'penetrating/crossing/permeating peptides' till July 2020, as an advanced search query that should be included in the research articles' title/abstract. | PubMed:(((Brain[Title/Abstract]) OR (blood–brain barrier[Title/Abstract])) AND peptide[Title/Abstract]) AND (transport[Title/Abstract] OR transfer[Title/Abstract] OR permeation[Title/Abstract] OR permeability[Title/Abstract])" , covering the period 2011.01–2021.11 |
| Negative sample search rules | UniProt with query "peptides length: [5 TO 50] NOT blood brain barrier NOT brain NOT permeation NOT permeability NOT brainpeps NOT transmembrane NOT transport NOT transfer NOT venom NOT toxin NOT membrane NOT neuro NOT hemolysis AND reviewed: yes". | Randomly generated 2690 non-BBPs from the Swiss-Prot database | UniProt with the query "peptides length: [5 TO 50] NOT blood brain barrier NOT brain NOT brainpeps NOT b3pdb NOT permeation NOT permeability NOT venom NOT toxin NOT transmembrane NOT transport NOT transfer NOT membrane NOT neuro NOT hemolysis AND reviewed: yes" |

| | | | |
|---|---|---|---|
| Article search deadline | / | 2020.07.22 | 2021.11 |
| Number of articles | 7 | 271 | 300 |
| Number of positive samples | 119 | 269 | 425 |
| Number of negative samples | 119 | 2690 | 425 |
| Peptide length | 5-50 | 6-30 | 5-50 |

Table S9 The prediction performances of the reproducibility analysis for nine classifiers

| Classifier | SN(%) | SP(%) | ACC(%) | MCC | AUC |
|---|---|---|---|---|---|
| RF | 75.36±5.54 | 77.14±4.62 | 76.25±3.56 | 0.5264±0.0710 | 0.8563±0.0309 |
| rbfSVM | 78.3±5.29 | 74.32±5.60 | 76.31±3.67 | 0.5283±0.0141 | 0.8300±0.0365 |
| KNN | 75.66±5.09 | 74.52±5.07 | 75.09±3.47 | 0.5032±0.0696 | 0.7509±0.0347 |
| DT | 68.9±6.95 | 67.36±5.88 | 68.13±4.35 | 0.3643±0.0876 | 0.6795±0.0507 |
| linearSVM | 66.68±6.52 | 74.74±5.30 | 70.71±4.24 | 0.4169±0.0848 | 0.7713±0.0433 |
| LSTM | 57.70±9.93 | 56.26±10.74 | 56.98±6.18 | 0.1410±0.1244 | 0.5795±0.0767 |
| AdaBoost | 64.96±5.97 | 71.40±6.33 | 68.18±4.33 | 0.3658±0.0872 | 0.7398±0.0435 |
| GentleBoost | 70.24±6.76 | 72.72±5.29 | 71.48±4.05 | 0.4314±0.0812 | 0.7730±0.0384 |
| LogitBoost | 69.46±6.26 | 73.96±5.49 | 71.71±3.41 | 0.4367±0.0684 | 0.7770±0.0393 |

## References


Cai, C.Z., Han, L.Y., Ji, Z.L., Chen, X., and Chen, Y.Z. (2003). SVM-Prot: Web-based support vector machine software for functional classification of a protein from its primary sequence. *Nucleic Acids Res* 31(13), 3692-3697. doi: 10.1093/nar/gkg600.

Cai, C.Z., Han, L.Y., Ji, Z.L., and Chen, Y.Z. (2004). Enzyme family classification by support vector machines. *Proteins* 55(1), 66-76. doi: 10.1002/prot.20045.

Chen, Z., Zhao, P., Li, F., Leier, A., Marquez-Lago, T.T., Wang, Y., et al. (2018). iFeature: a Python package and web server for features extraction and selection from protein and peptide sequences. *Bioinformatics* 34(14), 2499-2502. doi: 10.1093/bioinformatics/bty140.

Chou, K.C. (2001). Prediction of protein cellular attributes using pseudo-amino acid composition. *Proteins* 43(3), 246-255. doi: 10.1002/prot.1035.

Chou, K.C. (2005). Using amphiphilic pseudo amino acid composition to predict enzyme subfamily classes. *Bioinformatics* 21(1), 10-19. doi: 10.1093/bioinformatics/bth466.

Dubchak, I., Muchnik, I., Holbrook, S.R., and Kim, S.H. (1995). Prediction of protein folding class using global description of amino acid sequence. *Proc Natl Acad Sci U S A* 92(19), 8700-8704. doi: 10.1073/pnas.92.19.8700.

Dubchak, I., Muchnik, I., Mayor, C., Dralyuk, I., and Kim, S.H. (1999). Recognition of a protein fold in the context of the Structural Classification of Proteins (SCOP) classification. *Proteins* 35(4), 401-407.

Feng, Z.P., and Zhang, C.T. (2000). Prediction of membrane protein types based on the hydrophobic index of amino acids. *J Protein Chem* 19(4), 269-275. doi: 10.1023/a:1007091128394.

Horne, D.S. (1988). Prediction of protein helix content from an autocorrelation analysis of sequence hydrophobicities. *Biopolymers* 27(3), 451-477. doi: 10.1002/bip.360270308.

Jiao, Y.S., and Du, P.F. (2016). Predicting Golgi-resident protein types using pseudo amino acid compositions: Approaches with positional specific physicochemical properties. *J Theor Biol* 391, 35-42. doi: 10.1016/j.jtbi.2015.11.009.

Saravanan, V., and Gautham, N. (2015). Harnessing Computational Biology for Exact Linear B-Cell Epitope Prediction: A Novel Amino Acid Composition-Based Feature Descriptor. *Omics* 19(10), 648-658. doi: 10.1089/omi.2015.0095.

Sokal, R.R., and Thomson, B.A. (2006). Population structure inferred by local spatial autocorrelation: an example from an Amerindian tribal population. *Am J Phys Anthropol* 129(1), 121-131. doi: 10.1002/ajpa.20250.

Xiao, N., Cao, D.S., Zhu, M.F., and Xu, Q.S. (2015). protr/ProtrWeb: R package and web server for generating various numerical representation schemes of protein sequences. *Bioinformatics* 31(11), 1857-1859. doi: 10.1093/bioinformatics/btv042.
